# Perceptions, attitudes and understanding of health professionals of interprofessional practice at a selected community health centre

**DOI:** 10.4102/phcfm.v13i1.2724

**Published:** 2021-04-22

**Authors:** Luzaan Kock, Nondwe B. Mlezana, José M. Frantz

**Affiliations:** 1 Department of Physiotherapy, Faculty of Community and Health Sciences, University of the Western Cape, Bellville, South Africa; 2 Department of Research and Innovation, Office of the Deputy Vice-Chancellor, University of the Western Cape, Bellville, South Africa

**Keywords:** primary healthcare, interprofessional practice, community health centre, healthcare service delivery, healthcare model

## Abstract

**Background:**

Despite being identified as a solution to the challenges related to healthcare service delivery, the incorporation of interprofessional practice (IPP) into clinical practice has been limited. To implement an interprofessional model of healthcare, successfully, health professionals need to have an understanding of IPP and its related content.

**Aim:**

The aim of this study was to explore and describe the health professionals’ perceptions, attitudes and understanding of IPP at a selected community health centre.

**Setting:**

This study was conducted at a primary healthcare facility in the Western Cape, South Africa.

**Methods:**

Ethical clearance and permission to conduct the study was obtained from all relevant stakeholders. Four focus group discussions were conducted with health professionals at the facility. Themes, codes and categories were highlighted from the transcripts of the audiotape-recorded data.

**Findings:**

The findings suggest that health professionals do not have an understanding of IPP, and are thus unable to apply it practically. The health professionals perceived certain healthcare processes in the facility as barriers to the integration of practices. In addition, the health professionals expressed the need for interprofessional relationships, creation of opportunities for IPP, and communication to transform the current practice.

**Conclusion:**

To implement IPP into this facility, effectively, the authors of this study recommend that facility management implement campaigns for and training on, the transition to IPP, staff induction programmes and regular meetings.

## Introduction

A primary health care (PHC) workforce requires a wide range of experts from various sectors to work together to respond to population health needs.^1^ Interprofessional practice (IPP) has been identified as a means to improve patient’s experience, improve population health outcomes, decrease healthcare cost and improve the work experience of health professionals.^2^ As a result, there has been a global shift to an interprofessional model of healthcare. To ensure preparedness for IPP, adequate in-service training is required for health professionals.^1^ Therefore, the incorporation of IPP into clinical practice requires the creation of opportunities, where health professionals could develop skills and knowledge for effective collaboration.

Interprofessional education (IPE) has been defined as a learning approach, which allows professionals to learn with, from, and about each other to improve collaboration.^3^ In addition, the WHO highlighted that IPE in health improved patient outcomes.^3^ Within this learning approach, the skills, knowledge and values required to collaborate with other health professionals in practice are developed and enhanced.^4^ Various strategies are used to implement IPE, as well as IPP, and focuses on one, or more of the interprofessional core competencies. Interprofessional core competencies are the enactment of knowledge, skills and attitude required to collaborate effectively.^5^ Interprofessional practice and interprofessional education are thus interdependent to ensure improved health service delivery to the population.^2^

However, various barriers to the successful adoption of the IPP approach to healthcare have been identified, which include, time constraints, poor financial support, relationship building, communication, health professional versus patient responsibility, and patient-centred versus disease-focused models of care.^6^ In addition, South Africa is faced with staff shortages at PHC level in the public healthcare sector.^7^ Primary health care facilities often have only one representative per discipline, which is often an employee, who services more than one facility; however, one representative per discipline could be used to start an interprofessional approach.

Although barriers should not be viewed as resistance, instead it could be used as a guide to the incorporation of healthcare models into the health service delivery.^8^ In order to develop appropriate strategies, it is imperative to understand how health professionals perceived the implementation of IPP. In a study conducted by Bierwas et al.,^9^ the participants displayed a positive attitude towards interprofessional learning; however, the execution into practice remained limited. The reported reason for the poor integration into practice included the limited or no understanding of IPP, IPE, as well as the IPE core competencies. The development and delivery of IPE is shaped by various mechanisms including staff training, managerial support, logistics and scheduling, and programme content.^3^ When the local context is considered in the development of the IPE programme, the areas that require support can be highlighted.^3^ Similarly, structured protocols, communication strategies, shared decision-making processes, and the environment at the facility influences how IPP can be introduced and executed.^3^ To develop an appropriate IPE/IPP programme at a health facility, the context of the facility needs to be understood. To make appropriate recommendations for the successful implementation of IPP at a healthcare facility, it is important to highlight the areas of support that the staff at the facility need. The aim of this study therefore was to explore and describe health professionals’ perceptions, attitudes and understanding of IPP at a PHC facility.

## Method

### Study design

The researchers employed an exploratory, descriptive, qualitative case study design with focus group discussions (FGD) to explore and describe the perceptions, understanding and attitudes of health professionals regarding IPP.^10^

### Setting

This current study was conducted across different departments, within one facility at the PHC level. The facility is a community health care (CHC) centre that operates in the Nyanga health district of the Metropole Region, Western Cape, South Africa. The Nyanga health district is one of 11 sub-districts of the Metropole region. This CHC serves an urban population that gains access to the facility through internal, external or self-referrals. The CHC consists of a 24-h trauma unit, 24-h midwife obstetric unit, and a clinic. The clinic delivers the full PHC package to the population, and consists of administrators, a team of family physicians, various levels of nursing staff, a radiography team, pharmacists and pharmacy assistants, and allied health professionals. The allied health professional team is comprised of a physiotherapist, a dietician, a social worker, healthcare promoters and a sessional occupational therapist.

### Study population

The target population for this current study included all health professionals and administrative staff members, who interacted with patients, requiring health services. The researchers explained the purpose of the study to the rehabilitation manager, who subsequently, disseminated the information to the various departments, for individuals to volunteer as participants. The sample consisted of 33 individuals who offered their informed, signed consent to participate in the FGDs.

### Data collection

The data collection method included four FGDs, conducted with the health professionals and administrative staff. Each FGD comprised of four to 10 participants, depending on the availability of the staff members. Before each FGD was conducted, the participants had to declare confidentiality of information shared in the group, be assured of anonymity when reporting, as well as acknowledge their right to withdraw from the study at any time. Permission to audiotape-record the FGDs was obtained from all the participants. A semi-structured interview schedule, consisting of open-ended questions, was used to explore the perceptions, attitudes and understanding of health professionals, regarding IPP. The broad question used at the start of each FGD was: ‘What is your understanding of interprofessional practice?’ The following prompts were used: ‘What are your views of IPP at primary health care level?’ and ‘How do you think IPP can be implemented at your facility?’

The FGDs were conducted in a private area at the CHC and each FGD lasted between 30 and 60 min. All the interviews were conducted in English, as the participants were fluent in the language. The recorded FGDs were transcribed verbatim. To ensure dependability, two researchers coded the transcripts. To record contextual impressions and insights, notes were taken throughout the process. Member checking, by debriefing with the participants after the FGD, was performed to ensure credibility and trustworthiness.

### Data analysis

Using the 6-step, thematic analysis of Braun and Clarke,^11^ the researchers analysed the transcribed voice recordings. Each transcript was read individually by two researchers and notes were made in the margins to highlight interesting codes. The researchers followed a deductive method of analysis, for categorisation into sub-themes. Sub-themes from all the transcripts were grouped into themes. All sub-themes are supported by quotes from the FGDs.

### Ethical considerations

Ethics approval was obtained from the University of the Western Cape Biomedical Research Ethics Committee (Ethics number – BM19/1/38), The Western Cape Department of Health, and the management, as well as the participants at the CHC.

## Findings

### Characteristics of the participants

The study sample comprised 33 participants from different departments at a single CHC. Table 1 represents the gender, years of experience in the public health sector and profession of the participants.

**TABLE 1:** Characteristics of the participants.

Characteristic	Category	Number
Gender	Male	9
	Female	24
Years of experience	0–10 years	22
	11–20 years	7
	Longer than 20 years	4
Profession	Physician	6
	Physiotherapist	1
	Administrative clerk	5
	Nurse	17
	Pharmacist	2
	Radiographer	1
	Social worker	1

### Main findings

The findings describe the perceptions, attitudes and understanding of health professionals regarding IPP. The subthemes and categories are presented in Table 2. Quotes to support these sub-themes are presented below.

**TABLE 2:** Themes, subthemes and categories.

Themes	Subthemes	Categories
Understanding	Defining IPP	Relationships
		Referrals
Perceptions	Current processes	Case dependent
	Barriers	Hierarchy
		Logistical challenges
		Infrastructural barriers
		Time constraints
		Administration
Attitudes	Resistance	Lack of patient follow-up
		No change in outcome
		Setting
	Implementation	Relationship
		Communication
		Opportunity

IPP, interprofessional practice.

#### Defining interprofessional practice

The health professionals defined IPP as a professional relationship between colleagues:

‘A doctor and a nurse working on, on a patient together and then the patient maybe will go for an X-ray.’ (FGD1, P5, February 2020)

‘… the working together of the different professions who have roles and expectations.’ (FGD4, P5)

‘… where you work within … with your colleagues.’ (FGD1, P5)

It was evident that participants were unable to provide a definition of IPP. Health professionals thus failed to apply IPP in practice. Referral to another health professional without interprofessional interaction could mistakenly be seen as IPP:

‘We basically refer to Physio or Social Worker for social issues.’ (FGD3, P4)

‘It’s easier to, to refer because I mean, it’s just submitting work over.’ (FGD2, P1)

‘… if we refer for relevant staff.’ (FGD3, P4)

#### Current interprofessional practice processes

At this facility, the processes deemed as IPP involved referrals between staff, and health professionals making decisions in isolation. The lack of understanding may reflect what the participants were currently observing at their facility. When asked about their perceptions of the current IPP process at their facility, the participants expressed that there were ongoing attempts to integrate practices:

‘Actually, we work together with the Doctors.’ (FGD1, P5)

‘We do work like this sometimes. It’s depends to that case.’ (FGD1, P1)

‘… as a team, doctor, nurse or all those that are there … you have a discuss about the patient.’ (FGD2, P2)

#### Barriers

When asked what they perceived to be the reasons for the lack of interprofessional interaction, the participants identified various barriers. Their reasons related to hierarchy and logistical arrangements:

‘Hierarchy sometimes ok, and I’m a Doctor, I’m a Nurse, I’m a Clerk, I’m a cleaner.’ (FGD4, P1)

‘You don’t know when, when is the Physio in the office.’ (FGD1, P2)

‘I’m not gonna walk to Physio and explain my situation and rush back.’ (FGD3, P3)

Some participants expressed time barriers and administration, as a major hindrance to the successful implementation of IPP at their facility:

‘I don’t think it can’t be done. I think it’s about the time being set aside for it.’ (FGD4, P4)

‘… time constrains. I have 6 minutes with a patient.’ (FGD3, P3)

‘Everyone has different times … you’re off on Wednesdays, he’s off on Fridays.’ (FGD3, P5)

#### Resistance

Based on their view of IPP, participants expressed a negative attitude towards the implementation of IPP in the current healthcare processes at their facility. Participants expressed that because of large patient numbers and incomplete patient information systems, health professionals are unable to conduct regular patient follow-up:

‘I think it’s kind of difficult to do here, because you see a patient once.’ (FGD3, P2)

‘And you’d rather have the patient coming back sooner, than they should.’ (FGD3, P3)

‘… if we can actually have working phones and working numbers for these patients … you check your results for a half an hour for all the patients. If it’s abnormal, you call the patient ….’ (FGD3, P4)

When probed about the implementation of IPP into their facility, participants highlighted the challenges of working in a PHC setting when compared to levels of care:

‘I think it mostly happens in big hospitals departments sit down with a patient and discuss the patient, but in such clinics as it gets referred …’ (FGD3, P2)

‘… this is not a hospital, you can’t do that.’ (FGD3, P4)

‘Because, keep in mind that Primary Health Care at the end especially O.P.D there is a certain target they must reach.’ (FGD1, P1)

Participants expressed that the challenges faced in PHC result in no change in outcome in practice:

‘We’ve got so much pressure on us that this doesn’t go to my head … that there is no point.’ (FGD 3, P1)

‘A representative for every department, every unit would be there to be able to meet … But that with the change of management it fell off.’ (FGD2, P1)

‘You’ve got all the best policies, but somebody needs to apply them.’ (FGD4, P1)

#### Implementation

When probed on what would be required to implement IPP, successfully, the health professionals expressed the need for relationships, communication and opportunity for IPP. Participants highlighted the need to have interprofessional and interdepartmental relationships:

‘… to introduce the other staff from the other department in the Nurses day.’ (FGD1, P3)

‘I actually spoke to the trauma manager. I said, “you did not orientate them there. You did not introduce them at X-rays.”’ (FGD2, P5)

‘Team-building sessions … I think we need to.’

In order to implement IPP, participants expressed the need for time to participate in opportunities for IPP. However, one participant explained that the current referral process was more time-consuming:

‘I think it’s about the time being set aside for it.’ (FGD4, P4)

‘But we don’t have that time to sit …’ (FGD2, P6)

‘Maybe I don’t even have to go through a lengthy process of filling in a referral form and all that. You can give in everything you have discussed with this patient, I mean it’s easier.’ (FGD2, P1)

Participants highlighted the importance of creating platforms that promote communication. One participant expressed that interdepartmental communication is compromised due to faulty telephones at this facility:

‘Pharmacy all the time you find that there is that, that thing that disturbs the telephone you can’t phone, you can’t phone from us.’ (FGD1, P1)

‘… we can have a multidisciplinary team meetings.’ (FGD2, P4)

‘So when we sit and discuss these things they will understand my mind, I will understand their mind but it does not happen.’ (FGD2, P2)

## Discussion

Based on the findings, the current situation at the facility and the recommendations to ensure successful implementation of IPP are discussed.

### The current situation

It is apparent from the data that the participants at this facility adopt a multidisciplinary approach to patient management and believe it to be the same as IPP. In a multidisciplinary team approach, health professionals work in parallel with clear roles and predetermined tasks.^12^ However, Korner continues to explain that a multidisciplinary approach encourages hierarchical lines of authority. Hierarchy is considered a barrier to effective teamwork between various professionals.^13^ The presence of hierarchical systems create controlled lines of communication, which delays decision-making.^13^ At the PHC level, the healthcare process of a patient relies on referrals mainly from physicians and professional nurses, to allied health professionals. This referral system encourages instruction, as opposed to collaboration. At this PHC facility, the hierarchical system is sustained through the referral process, as the physician makes referrals without communication or interaction with other health professionals. In order to combat the current hierarchies at this facility, hierarchical systems, such as referrals without interprofessional interaction need to be reconsidered. It is thus noted that interprofessional interactions amongst staff members at this facility is required.

However, the current logistical and infrastructural situation of this facility does not allow for interprofessional interaction. One participant expressed the inconvenience of referring the patient to a professional in another department. The participants highlighted faulty telephone lines as a reason for the decreased staff interaction. Often, the departments in a PHC facility are spaced too far apart, making it time-consuming to communicate with professionals from other departments without functional communication technology. Time constraints has been highlighted as a barrier to the successful implementation of IPP.^6^ It is important to note that sufficient time is required to ensure effective communication, and overcome prejudices between health professionals.^6^

### Recommendations for the successful implementation of interprofessional practice

Interpofessional practice can be used to improve the work environment of health professionals.^2^ It is thus noted that participants highlighted the need for an interprofessional relationship, opportunities for IPP, and communication. Recommendations for the successful implementation include IPP opportunities that will most likely result in an improved interprofessional relationship and communication. Staff induction programmes reduce ambiguity, results in role clarity and facilitates the ability of new staff members to comprehend the process of their new environment.^14^ The implementation of staff induction programmes could be used effectively to develop, or enhance, the role clarification competency needed for effective collaboration.^5^ Role clarification is the ability of individuals to describe their own role, as well as the role of other health professionals.^15^

Staff expressed the need for opportunities for IPP. However, given the lack of staff interaction, when one staff member is away on leave, it is unlikely that staff in other departments would be aware of it. As IPP depends on the presence of various professional staff, it is important to create platforms of open discussions. In these opportunities, staff could indicate when they are on annual or sick leave, working and break times, or attending to organisational responsibilities. To ensure the representation of various professionals in IPP interventions, facility management should ensure the development of regular interaction between departments.

Improving communication is essential in the transformation to high quality care.^16^ Improving communication is perceived as an essential area for team-training.^17^ Opportunities to encourage interprofessional communication need to be created. To ensure that interprofessional interaction does not result in a negative impact on service delivery, these opportunities need to be efficient and have pre-determined time limits. By incorporating short, regular interprofessional meetings into the practice at PHC level, health professionals could discuss interventions that the patient received, prior to referral.^18^ Regular meetings are required to develop and improve collaboration.^18^ Regular meetings may lead to more flexible interaction between health professionals, as it encourages communication and contact.^18^ In addition, regular meetings may encourage discussion on team expectations.^18^

### Strengths and limitations

The findings of this current study cannot be generalised for all health professionals rendering services at the PHC level. The findings, however, could assist in gaining insight to the experiences of health professionals at the PHC level in the Cape metropole SA. Given the time constraints of the FGD, there was an impact on the depth of analysis of this study. The members were able to debrief with participants after the session.

### Implications of the study

By creating staff training opportunities that promote interprofessional relationships and interprofessional communication, staff may develop a positive attitude towards the transition to an interprofessional model of care.

## Conclusion

This study found that health professionals at this facility do not have an understanding of IPP, and are therefore unaware as to how to engage in IPP. To ensure the integration of care, the authors of this current study recommend that facility management hosts awareness campaigns, regarding the transition from referral to collaboration. Should the management want to implement IPP into this PHC facility effectively, the implementation of staff induction programmes and regular interprofessional meetings is recommended.
